# Diaqua­bis(5-bromo-2-hydroxy­benzoato)bis­(*N*-methyl­nicotinamide)zinc(II)

**DOI:** 10.1107/S1600536810008834

**Published:** 2010-03-13

**Authors:** Zuzana Bujdošová, Juraj Kuchár, Katarína Györyová

**Affiliations:** a Department of Inorganic Chemistry, Institute of Chemistry, P. J. Šafárik University, Moyzesova 11, 041 54 Košice, Slovakia

## Abstract

The title mononuclear complex mol­ecule, [Zn(C_7_H_4_BrO_3_)_2_(C_7_H_8_N_2_O)_2_(H_2_O)_2_], has a crystallographically imposed centre of symmetry. The zinc(II) atom is coordinated by two N atoms from two *N*-methyl­nicotinamide ligands, two O atoms from two 5-bromo­salicylate anions and two aqua O atoms in a slightly distorted octa­hedral geometry. Intra­molecular O—H⋯O hydrogen-bonding inter­actions are present. In the crystal structure, mol­ecules are linked by inter­molecular O—H⋯O and N—H⋯O hydrogen bonds, forming a two-dimensional network perpendicular to [100].

## Related literature

For general background to the properties of carboxylic acid–metal complexes, see: Nagar (1990 ▶); Cavagiolio *et al.* (2000 ▶). For the synthesis and properties of zinc(II) carboxyl­ates reported by our group, see: Györyová *et al.* (2005 ▶, 2006 ▶); Bujdošová *et al.* (2009 ▶); Gebicki *et al.* (2003 ▶). For related structures, see: Necefoglu *et al.* (2001*a* ▶,*b* ▶); Hökelek *et al.* (2007 ▶, 2009*a* ▶,*b* ▶); Öztürk *et al.* (2008 ▶); Sarı *et al.* (2007 ▶); Liu *et al.* (2004 ▶). 
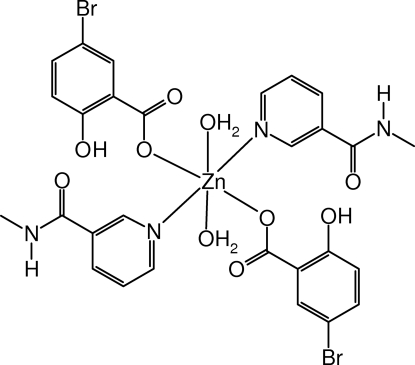


## Experimental

### 

#### Crystal data

[Zn(C_7_H_4_BrO_3_)_2_(C_7_H_8_N_2_O)_2_(H_2_O)_2_]
*M* *_r_* = 805.73
Triclinic, 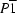

*a* = 8.1600 (2) Å
*b* = 10.1122 (3) Å
*c* = 10.4291 (3) Å
α = 66.800 (3)°
β = 74.334 (2)°
γ = 80.743 (2)°
*V* = 760.15 (4) Å^3^
*Z* = 1
Mo *K*α radiation
μ = 3.50 mm^−1^
*T* = 290 K
0.57 × 0.30 × 0.26 mm

#### Data collection

Oxford Diffraction Xcalibur Sapphire2 diffractometer
Absorption correction: numerical [Clark & Reid (1995 ▶) in *CrysAlis PRO* (Oxford Diffraction, 2009 ▶)] *T*_min_ = 0.289, *T*_max_ = 0.484
32240 measured reflections
3153 independent reflections
2686 reflections with *I* > 2σ(*I*)
*R*_int_ = 0.024

#### Refinement

*R*[*F* ^2^ > 2σ(*F* ^2^)] = 0.022
*wR*(*F* ^2^) = 0.064
*S* = 1.15
3153 reflections
195 parameters
H-atom parameters constrained
Δρ_max_ = 0.50 e Å^−3^
Δρ_min_ = −0.49 e Å^−3^

### 

Data collection: *CrysAlis PRO* (Oxford Diffraction, 2009 ▶); cell refinement: *CrysAlis PRO*; data reduction: *CrysAlis PRO*; program(s) used to solve structure: *SHELXS97* (Sheldrick, 2008 ▶); program(s) used to refine structure: *SHELXL97* (Sheldrick, 2008 ▶); molecular graphics: *DIAMOND* (Crystal Impact, 2007 ▶); software used to prepare material for publication: *SHELXL97*.

## Supplementary Material

Crystal structure: contains datablocks I, global. DOI: 10.1107/S1600536810008834/rz2423sup1.cif

Structure factors: contains datablocks I. DOI: 10.1107/S1600536810008834/rz2423Isup2.hkl

Additional supplementary materials:  crystallographic information; 3D view; checkCIF report

## Figures and Tables

**Table 1:** Hydrogen-bond geometry (Å, °)

*D*—H⋯*A*	*D*—H	H⋯*A*	*D*⋯*A*	*D*—H⋯*A*
O3—H1*O*3⋯O2	0.82	1.81	2.540 (2)	147
N2—H1*N*2⋯O3^i^	0.86	2.55	3.1109 (19)	123
O5—H2*O*5⋯O2^ii^	0.82	1.88	2.6694 (18)	162
O5—H1*O*5⋯O4^iii^	0.82	1.94	2.7568 (13)	179

Symmetry codes: (i) 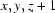; (ii) 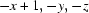; (iii) 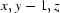.

**Fig. 1.**
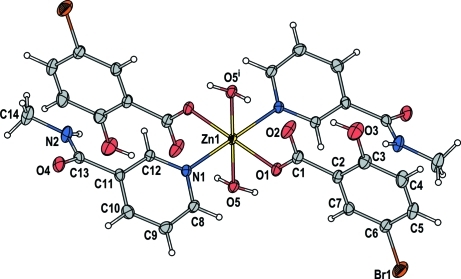
The molecular structure of the title compound with the atom-numbering scheme. Displacement ellipsoids are drawn at the 30% probability level. Symmetry code: (i) 1-x, -y, -z.

**Fig. 2.**
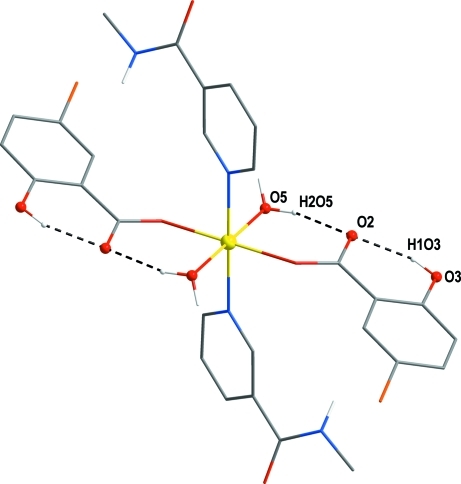
View of the intramolecular hydrogen bonds (dashed lines) of the title compound. Hydrogen atoms of aromatic rings and methyl groups are omitted for clarity.

**Fig. 3.**
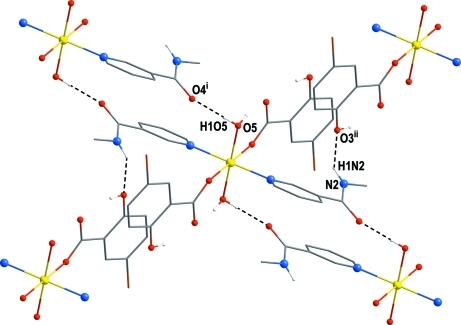
View of the intermolecular hydrogen bonds (dashed lines) of the title compound. Hydrogen atoms of aromatic rings and methyl groups are omitted for clarity. Symmetry codes: (i) x, -1+y, z; (ii) x, y, 1+z

## supplementary crystallographic information

### Comment

The complexes of carboxylic acids with metals, e.g. zinc, are interesting due to different coordination modes of a carboxylate group bound to a metal ion. It is well documented that heterocyclic compounds, especially N-donor ligand systems, play a significant role in many biological systems, being a component of several vitamins and drugs (Nagar, 1990; Cavagiolio *et al.*, 2000). As a part of our ongoing studies of zinc(II) carboxylates (Györyová *et al.*, 2005; Györyová *et al.*, 2006; Bujdošová *et al.*, 2009) we have been exploring the synthesis and crystal structure of zinc(II) 5-bromosalicylate containing *N*-methylnicotinamide, shown at in vitro study to be a potent anti-inflammatory agent (Gebicki *et al.*, 2003).

In the title monomeric complex [Zn(C_7_H_4_BrO_3_)_2_(C_7_H_8_N_2_O)_2_(H_2_O)_2_] (Fig. 1), the zinc(II) atom, which lies on an inversion centre, exhibits a slightly distorted octahedral coordination geometry. The coordination sphere consists of three pairs of *trans*-arranged monodentate ligands. The two *N*-methylnicotinamide ligands are coordinated to the zinc atom through nitrogen atoms of the pyridine rings. The 5-bromosalicylate anion is coordinated through one oxygen atom of the carboxylate group. The similar distances O1—C1 (1.256 (2) Å) and O2—C1 (1.262 (2) Å) in the carboxylate group indicate a delocalized bonding arrangement and may be compared with the corresponding distances found in [Zn(C_7_H_4_ClO_2_)_2_(C_10_H_14_N_2_O)_2_(H_2_O)_2_] (Sarı *et al.*, 2007). The plane of the carboxylate group is approximately coplanar with the plane of the benzene ring; the dihedral angle between these planes is 5.5 (3)°. Such behaviour is not unusual; a similar arrangement was observed in other compounds (e.g. diaquabis(*N*,*N*-diethylnicotinamide-*N*)bis(4-fluorobenzoato-O)zinc(II) (Hökelek *et al.*, 2007)). All other geometric parameters of the coordinated anion are similar to that found in free 5-bromosalicylic acid (Liu *et al.*, 2004). The distorted octahedral coordination is completed by two pyridine nitrogen atoms of two *N*-methylnicotinamide ligands in the axial positions. The Zn—N distance (2.1583 (9) Å) is in good agreement with the values reported for other octahedrally coordinated zinc(II) complexes [viz., Diaquabis(4-chlorobenzoato)bis(*N*,*N*-diethylnicotinamide)zinc(II), Zn—N: 2.157 (3) Å; Sarı *et al.*, 2007]. The coordination environment of the zinc(II) atom is completed by water molecules, forming with carboxylate oxygen atoms the basal plane of the distorted octahedron. The Zn—O distance (2.1396 (8) Å) is comparable with those found in similar compounds [viz. diaquabis(2-bromobenzoato)bis(*N*,*N*-diethylnicotinamide)zinc(II), Zn—O: 2.1269 (12) Å; Hökelek *et al.*, 2009b]. Intramolecular hydrogen bonding interactions involving the hydroxyl groups and carboxylate oxygen atoms (O3—H1O3···O2) and the equatorially coordinated water molecule and carboxylate oxygen atom (O5—H2O5···O2) stabilize the molecular structure (Fig. 2). Intramolecular hydrogen bonds also influences the orientation and delocalized character of carboxylate group. The molecules of the title compound are linked into a two-dimensional network perpendicular to [100] by intermolecular O5—H1O5···O4 and N2—H1N2···O3 hydrogen bonds (Fig. 3).

### Experimental

Analytical reagent grade chemicals were used for the preparation of the title compound. The synthesis was carried out by reaction of aqueous solutions (20 ml) of ZnCl_2_ (0.14 g, 1 mmol) and NaHCO_3_ (0.17 g, 2 mmol). After complete removal of chloride anions, an acetone solution (10 ml) of 5-bromosalicylic acid (0.44 g, 2 mmol) was added. The resulting solution of (5-BrC_6_H_3_-2-(OH)COO)_2_Zn (0.50 g, 1 mmol) was mixed with an aqueous solution (10 ml) of *N*-methylnicotinamide (0.27 g, 2 mmol). The reaction mixture was stirred for 2 h and left aside for crystallization at room temperature. After two days, a small amount of colourless bright crystals appeared. The resulting crystals were isolated by filtration.

### Refinement

The hydrogen atoms of the water molecule were located in difference Fourier map and refined with the O—H distances constrained to 0.82 Å and with U_iso_(H) = 1.5U_eq_(O). The H atom bound to N2 was located in a difference Fourier map and refined U_iso_(H) = 1.2U_eq_(N). All other H atoms were positioned geometrically and constrained to ride on their parent atoms, with O—H = 0.82 Å, C—H = 0.93–0.96 Å, and with U_iso_(H) = 1.2U_eq_(C) or 1.5U_eq_(C, O) for methyl and hydroxyl H atoms.

### Crystal data

**Table:**

[Zn(C_7_H_4_BrO_3_)_2_(C_7_H_8_N_2_O)_2_(H_2_O)_2_]	*Z* = 1
*M**_r_* = 805.73	*F*(000) = 404
Triclinic, *P*1	*D*_x_ = 1.760 Mg m^−^^3^
Hall symbol: -P 1	Mo *K*α radiation, λ = 0.71073 Å
*a* = 8.1600 (2) Å	Cell parameters from 19248 reflections
*b* = 10.1122 (3) Å	θ = 3.0–29.6°
*c* = 10.4291 (3) Å	µ = 3.50 mm^−^^1^
α = 66.800 (3)°	*T* = 290 K
β = 74.334 (2)°	Prism, colourless
γ = 80.743 (2)°	0.56 × 0.30 × 0.26 mm
*V* = 760.15 (4) Å^3^	

### Data collection

**Table:**

Oxford Diffraction Xcalibur Sapphire2 diffractometer	3153 independent reflections
Radiation source: Enhance (Mo) X-ray Source	2686 reflections with *I* > 2σ(*I*)
graphite	*R*_int_ = 0.024
Detector resolution: 8.3438 pixels mm^-1^	θ_max_ = 26.5°, θ_min_ = 3.0°
ω scans	*h* = −10→10
Absorption correction: numerical [Clark & Reid (1995) in *CrysAlis PRO* (Oxford Diffraction, 2009)]	*k* = −12→12
*T*_min_ = 0.289, *T*_max_ = 0.484	*l* = −13→13
32240 measured reflections	

### Refinement

**Table:**

Refinement on *F*^2^	Primary atom site location: structure-invariant direct methods
Least-squares matrix: full	Secondary atom site location: difference Fourier map
*R*[*F*^2^ > 2σ(*F*^2^)] = 0.022	Hydrogen site location: inferred from neighbouring sites
*wR*(*F*^2^) = 0.064	H-atom parameters constrained
*S* = 1.15	*w* = 1/[σ^2^(*F*_o_^2^) + (0.0404*P*)^2^] where *P* = (*F*_o_^2^ + 2*F*_c_^2^)/3
3153 reflections	(Δ/σ)_max_ < 0.001
195 parameters	Δρ_max_ = 0.50 e Å^−^^3^
0 restraints	Δρ_min_ = −0.49 e Å^−^^3^

### Special details

**Table:**

Geometry. All esds (except the esd in the dihedral angle between two l.s. planes) are estimated using the full covariance matrix. The cell esds are taken into account individually in the estimation of esds in distances, angles and torsion angles; correlations between esds in cell parameters are only used when they are defined by crystal symmetry. An approximate (isotropic) treatment of cell esds is used for estimating esds involving l.s. planes.
Refinement. Refinement of *F*^2^ against ALL reflections. The weighted *R*-factor wR and goodness of fit *S* are based on *F*^2^, conventional *R*-factors *R* are based on *F*, with *F* set to zero for negative *F*^2^. The threshold expression of *F*^2^ > σ(*F*^2^) is used only for calculating *R*-factors(gt) etc. and is not relevant to the choice of reflections for refinement. *R*-factors based on *F*^2^ are statistically about twice as large as those based on *F*, and *R*- factors based on ALL data will be even larger.

### Fractional atomic coordinates and isotropic or equivalent isotropic displacement parameters (Å^2^)

**Table:**

	*x*	*y*	*z*	*U*_iso_*/*U*_eq_	
Zn1	0.5000	0.0000	0.0000	0.02312 (9)	
O1	0.67676 (14)	−0.02707 (13)	−0.17587 (12)	0.0305 (3)	
O2	0.5864 (2)	0.14251 (15)	−0.36177 (15)	0.0493 (4)	
O3	0.6562 (2)	0.11216 (16)	−0.60156 (15)	0.0550 (4)	
H1O3	0.6075	0.1466	−0.5409	0.083*	
C1	0.6699 (2)	0.02704 (19)	−0.30545 (19)	0.0299 (4)	
C2	0.7659 (2)	−0.05359 (18)	−0.40050 (18)	0.0270 (4)	
C3	0.7497 (2)	−0.00760 (19)	−0.54230 (19)	0.0348 (4)	
C4	0.8343 (3)	−0.0873 (2)	−0.6265 (2)	0.0425 (5)	
H4	0.8233	−0.0574	−0.7204	0.051*	
C5	0.9340 (3)	−0.2099 (2)	−0.5713 (2)	0.0403 (5)	
H5	0.9899	−0.2629	−0.6276	0.048*	
C6	0.9505 (2)	−0.25379 (19)	−0.43114 (19)	0.0319 (4)	
C7	0.8667 (2)	−0.17721 (18)	−0.34631 (18)	0.0295 (4)	
H7	0.8777	−0.2085	−0.2522	0.035*	
Br1	1.08861 (3)	−0.42202 (2)	−0.35493 (2)	0.05222 (9)	
N1	0.66055 (11)	0.14385 (9)	0.01039 (9)	0.0245 (3)	
N2	0.48397 (11)	0.41145 (9)	0.24800 (9)	0.0384 (4)	
H1N2	0.4560	0.3237	0.2876	0.046*	
C8	0.82847 (11)	0.13682 (9)	−0.04349 (9)	0.0302 (4)	
H8	0.8741	0.0682	−0.0843	0.036*	
C9	0.9368 (2)	0.2262 (2)	−0.0414 (2)	0.0373 (4)	
H9	1.0533	0.2178	−0.0794	0.045*	
C10	0.8694 (2)	0.32926 (19)	0.0186 (2)	0.0352 (4)	
H10	0.9399	0.3919	0.0205	0.042*	
C11	0.6960 (2)	0.33753 (16)	0.07550 (17)	0.0250 (3)	
C12	0.5966 (2)	0.24263 (16)	0.06900 (17)	0.0241 (3)	
H12	0.4799	0.2479	0.1072	0.029*	
C13	0.6211 (2)	0.44766 (17)	0.14122 (19)	0.0282 (4)	
C14	0.39754 (10)	0.50534 (8)	0.32554 (9)	0.0531 (6)	
H14C	0.2890	0.4692	0.3828	0.080*	
H14A	0.3809	0.6011	0.2583	0.080*	
H14B	0.4660	0.5074	0.3864	0.080*	
O4	0.68500 (13)	0.56491 (9)	0.09551 (11)	0.0373 (3)	
O5	0.63493 (12)	−0.17592 (8)	0.13428 (10)	0.0300 (3)	
H2O5	0.5760	−0.1834	0.2138	0.045*	
H1O5	0.6509	−0.2531	0.1229	0.045*	

### Atomic displacement parameters (Å^2^)

**Table:**

	*U*^11^	*U*^22^	*U*^33^	*U*^12^	*U*^13^	*U*^23^
Zn1	0.02369 (15)	0.02253 (14)	0.02842 (16)	−0.00151 (10)	−0.00284 (11)	−0.01690 (11)
O1	0.0301 (6)	0.0377 (7)	0.0285 (7)	−0.0004 (5)	−0.0009 (5)	−0.0214 (6)
O2	0.0659 (10)	0.0349 (7)	0.0386 (8)	0.0174 (7)	−0.0051 (7)	−0.0168 (6)
O3	0.0737 (11)	0.0479 (9)	0.0376 (9)	0.0209 (8)	−0.0187 (8)	−0.0152 (7)
C1	0.0294 (9)	0.0296 (9)	0.0322 (10)	−0.0047 (7)	0.0013 (7)	−0.0175 (8)
C2	0.0290 (9)	0.0285 (9)	0.0242 (9)	−0.0033 (7)	0.0008 (7)	−0.0143 (7)
C3	0.0416 (11)	0.0330 (10)	0.0272 (9)	−0.0004 (8)	−0.0060 (8)	−0.0102 (8)
C4	0.0580 (13)	0.0465 (12)	0.0239 (10)	0.0003 (10)	−0.0069 (9)	−0.0170 (9)
C5	0.0489 (12)	0.0433 (11)	0.0322 (10)	−0.0008 (9)	0.0002 (9)	−0.0246 (9)
C6	0.0315 (10)	0.0310 (9)	0.0344 (10)	0.0009 (7)	−0.0031 (8)	−0.0172 (8)
C7	0.0321 (9)	0.0342 (9)	0.0247 (9)	−0.0028 (7)	−0.0033 (7)	−0.0152 (7)
Br1	0.05614 (16)	0.04580 (14)	0.05557 (16)	0.01938 (10)	−0.01506 (11)	−0.02663 (11)
N1	0.0260 (7)	0.0214 (7)	0.0303 (8)	−0.0008 (5)	−0.0066 (6)	−0.0140 (6)
N2	0.0486 (10)	0.0283 (8)	0.0416 (10)	−0.0049 (7)	−0.0014 (8)	−0.0213 (7)
C8	0.0287 (9)	0.0277 (9)	0.0376 (10)	0.0011 (7)	−0.0036 (7)	−0.0192 (8)
C9	0.0248 (9)	0.0371 (10)	0.0539 (12)	−0.0040 (8)	−0.0013 (8)	−0.0249 (9)
C10	0.0346 (10)	0.0300 (9)	0.0462 (11)	−0.0087 (7)	−0.0085 (8)	−0.0173 (8)
C11	0.0327 (9)	0.0181 (8)	0.0272 (9)	−0.0012 (6)	−0.0100 (7)	−0.0097 (7)
C12	0.0262 (8)	0.0215 (8)	0.0273 (9)	0.0006 (6)	−0.0069 (7)	−0.0122 (7)
C13	0.0331 (9)	0.0229 (8)	0.0359 (10)	0.0034 (7)	−0.0163 (7)	−0.0151 (7)
C14	0.0612 (15)	0.0488 (13)	0.0547 (14)	−0.0050 (11)	0.0057 (11)	−0.0365 (11)
O4	0.0450 (8)	0.0217 (6)	0.0504 (8)	−0.0032 (5)	−0.0097 (6)	−0.0191 (6)
O5	0.0316 (7)	0.0239 (6)	0.0381 (7)	0.0014 (5)	−0.0059 (5)	−0.0175 (5)

### Geometric parameters (Å, °)

**Table:**

Zn1—O1^i^	2.0879 (11)	N1—C8	1.3352 (13)
Zn1—O1	2.0879 (11)	N1—C12	1.3359 (17)
Zn1—O5	2.1396 (9)	N2—C13	1.328 (2)
Zn1—O5^i^	2.1396 (9)	N2—C14	1.4609 (13)
Zn1—N1^i^	2.1583 (9)	N2—H1N2	0.8578
Zn1—N1	2.1583 (9)	C8—C9	1.372 (2)
O1—C1	1.256 (2)	C8—H8	0.9300
O2—C1	1.262 (2)	C9—C10	1.387 (2)
O3—C3	1.341 (2)	C9—H9	0.9300
O3—H1O3	0.8200	C10—C11	1.382 (2)
C1—C2	1.508 (2)	C10—H10	0.9300
C2—C7	1.386 (2)	C11—C12	1.384 (2)
C2—C3	1.402 (3)	C11—C13	1.497 (2)
C3—C4	1.396 (2)	C12—H12	0.9300
C4—C5	1.377 (3)	C13—O4	1.235 (2)
C4—H4	0.9300	C14—H14C	0.9600
C5—C6	1.389 (3)	C14—H14A	0.9600
C5—H5	0.9300	C14—H14B	0.9600
C6—C7	1.376 (2)	O5—H2O5	0.8196
C6—Br1	1.8968 (18)	O5—H1O5	0.8197
C7—H7	0.9300		
			
O1^i^—Zn1—O1	180.00 (8)	C6—C7—H7	119.9
O1^i^—Zn1—O5	92.30 (4)	C2—C7—H7	119.9
O1—Zn1—O5	87.70 (4)	C8—N1—C12	117.96 (11)
O1^i^—Zn1—O5^i^	87.70 (4)	C8—N1—Zn1	120.28 (7)
O1—Zn1—O5^i^	92.30 (4)	C12—N1—Zn1	121.77 (9)
O5—Zn1—O5^i^	180.00 (10)	C13—N2—C14	122.98 (11)
O1^i^—Zn1—N1^i^	91.23 (4)	C13—N2—H1N2	120.1
O1—Zn1—N1^i^	88.77 (4)	C14—N2—H1N2	115.5
O5—Zn1—N1^i^	91.78 (3)	N1—C8—C9	122.98 (11)
O5^i^—Zn1—N1^i^	88.22 (3)	N1—C8—H8	118.5
O1^i^—Zn1—N1	88.77 (4)	C9—C8—H8	118.5
O1—Zn1—N1	91.23 (4)	C8—C9—C10	118.76 (15)
O5—Zn1—N1	88.22 (4)	C8—C9—H9	120.6
O5^i^—Zn1—N1	91.78 (3)	C10—C9—H9	120.6
N1^i^—Zn1—N1	180.00 (4)	C11—C10—C9	118.99 (16)
C1—O1—Zn1	128.42 (11)	C11—C10—H10	120.5
C3—O3—H1O3	109.5	C9—C10—H10	120.5
O1—C1—O2	125.03 (16)	C10—C11—C12	118.22 (15)
O1—C1—C2	117.54 (15)	C10—C11—C13	119.77 (15)
O2—C1—C2	117.42 (16)	C12—C11—C13	122.01 (15)
C7—C2—C3	119.42 (15)	N1—C12—C11	123.07 (14)
C7—C2—C1	119.96 (15)	N1—C12—H12	118.5
C3—C2—C1	120.57 (16)	C11—C12—H12	118.5
O3—C3—C4	118.17 (17)	O4—C13—N2	123.41 (16)
O3—C3—C2	122.26 (16)	O4—C13—C11	120.51 (15)
C4—C3—C2	119.57 (17)	N2—C13—C11	116.08 (15)
C5—C4—C3	120.41 (18)	N2—C14—H14C	109.5
C5—C4—H4	119.8	N2—C14—H14A	109.5
C3—C4—H4	119.8	H14C—C14—H14A	109.5
C4—C5—C6	119.57 (17)	N2—C14—H14B	109.5
C4—C5—H5	120.2	H14C—C14—H14B	109.5
C6—C5—H5	120.2	H14A—C14—H14B	109.5
C7—C6—C5	120.74 (17)	Zn1—O5—H2O5	100.9
C7—C6—Br1	119.51 (14)	Zn1—O5—H1O5	119.2
C5—C6—Br1	119.74 (14)	H2O5—O5—H1O5	111.8
C6—C7—C2	120.28 (16)		
			
O5—Zn1—O1—C1	167.76 (16)	O1—Zn1—N1—C8	−24.26 (8)
O5^i^—Zn1—O1—C1	−12.24 (16)	O5—Zn1—N1—C8	63.39 (8)
N1^i^—Zn1—O1—C1	75.93 (14)	O5^i^—Zn1—N1—C8	−116.61 (8)
N1—Zn1—O1—C1	−104.07 (14)	O1^i^—Zn1—N1—C12	−24.81 (11)
Zn1—O1—C1—O2	24.7 (3)	O1—Zn1—N1—C12	155.19 (11)
Zn1—O1—C1—C2	−153.98 (11)	O5—Zn1—N1—C12	−117.16 (10)
O1—C1—C2—C7	−4.4 (2)	O5^i^—Zn1—N1—C12	62.84 (10)
O2—C1—C2—C7	176.77 (16)	C12—N1—C8—C9	−0.15 (17)
O1—C1—C2—C3	173.34 (16)	Zn1—N1—C8—C9	179.32 (11)
O2—C1—C2—C3	−5.5 (3)	N1—C8—C9—C10	−0.4 (2)
C7—C2—C3—O3	−178.98 (17)	C8—C9—C10—C11	0.7 (3)
C1—C2—C3—O3	3.3 (3)	C9—C10—C11—C12	−0.5 (3)
C7—C2—C3—C4	0.4 (3)	C9—C10—C11—C13	179.74 (17)
C1—C2—C3—C4	−177.40 (16)	C8—N1—C12—C11	0.3 (2)
O3—C3—C4—C5	179.01 (19)	Zn1—N1—C12—C11	−179.13 (12)
C2—C3—C4—C5	−0.4 (3)	C10—C11—C12—N1	0.0 (2)
C3—C4—C5—C6	−0.2 (3)	C13—C11—C12—N1	179.73 (14)
C4—C5—C6—C7	0.8 (3)	C14—N2—C13—O4	−2.0 (2)
C4—C5—C6—Br1	−179.66 (15)	C14—N2—C13—C11	178.73 (11)
C5—C6—C7—C2	−0.8 (3)	C10—C11—C13—O4	31.6 (2)
Br1—C6—C7—C2	179.66 (13)	C12—C11—C13—O4	−148.18 (17)
C3—C2—C7—C6	0.2 (3)	C10—C11—C13—N2	−149.10 (16)
C1—C2—C7—C6	178.00 (16)	C12—C11—C13—N2	31.1 (2)
O1^i^—Zn1—N1—C8	155.74 (8)		

Symmetry codes: (i) −*x*+1, −*y*, −*z*.

### Hydrogen-bond geometry (Å, °)

**Table:**

*D*—H···*A*	*D*—H	H···*A*	*D*···*A*	*D*—H···*A*
O3—H1O3···O2	0.82	1.81	2.540 (2)	147
N2—H1N2···O3^ii^	0.86	2.55	3.1109 (19)	123
O5—H2O5···O2^i^	0.82	1.88	2.6694 (18)	162
O5—H1O5···O4^iii^	0.82	1.94	2.7568 (13)	179

Symmetry codes: (ii) *x*, *y*, *z*+1; (i) −*x*+1, −*y*, −*z*; (iii) *x*, *y*−1, *z*.
